# Serosurvey of Human Antibodies Recognizing *Aedes aegypti* D7 Salivary Proteins in Colombia

**DOI:** 10.3389/fpubh.2018.00111

**Published:** 2018-05-18

**Authors:** Berlin L. Londono-Renteria, Heman Shakeri, Paula Rozo-Lopez, Michael J. Conway, Natasha Duggan, Majid Jaberi-Douraki, Tonya M. Colpitts

**Affiliations:** ^1^Department of Entomology, Kansas State University, Manhattan, KS, United States; ^2^Department of Anatomy and Physiology, Institute of Computational Comparative Medicine, Kansas State University, Manhattan, KS, United States; ^3^Central Michigan University College of Medicine, Mount Pleasant, MI, United States; ^4^Department of Cell Biology, University of Miami Miller School of Medicine, Miami, FL, United States; ^5^Department of Mathematics, Institute of Computational Comparative Medicine, Kansas State University, Manhattan, KS, United States; ^6^National Emerging Infectious Diseases Laboratories (NEIDL), Department of Microbiology, Boston University School of Medicine, Boston, MA, United States

**Keywords:** *Aedes* saliva, D7, antibody levels, biomarker, dengue

## Abstract

**Background:**

Dengue is one of the most geographically significant mosquito-borne viral diseases transmitted by *Aedes* mosquitoes. During blood feeding, mosquitoes deposit salivary proteins that induce antibody responses. These can be related to the intensity of exposure to bites. Some mosquito salivary proteins, such as D7 proteins, are known as potent allergens. The antibody response to D7 proteins can be used as a marker to evaluate the risk of exposure and disease transmission and provide critical information for understanding the dynamics of vector–host interactions.

**Methods:**

The study was conducted at the Los Patios Hospital, Cucuta, Norte de Santander, Colombia. A total of 63 participants were enrolled in the study. Participants were categorized into three disease status groups, age groups, and socioeconomic strata. The level of IgG antibodies against D7 *Aedes* proteins was determined by ELISA. We used a statistical approach to determine if there is an association between antibody levels and factors such as age, living conditions, and dengue virus (DENV) infection.

**Results:**

We found that IgG antibodies against D7 proteins were higher in non-DENV infected individuals in comparison to DENV-infected participants. Also, the age factor showed a significant positive correlation with IgG antibodies against D7 proteins, and the living conditions (socioeconomic stratification), in people aged 20 years or older, are a statistically significant factor in the variability of IgG antibodies against D7 proteins.

**Conclusion:**

This pilot study represents the first approximation to elucidate any correlation between the antibody response against mosquito D7 salivary proteins and its correlation with age, living conditions, and DENV infection in a dengue endemic area.

## Introduction

Dengue is one of the most globally significant mosquito-borne viral diseases, and epidemics are often recorded unevenly across socioeconomic strata (1). Tropical and subtropical areas of the Americas have shown a significant rise in dengue virus (DENV) transmission in recent years; especially in South America, where more than 80% of the population lives in areas of high risk (2). Historically, Colombia is one of the countries most affected by epidemics of dengue disease (3). Colombia has hyper-endemicity of multiple DENV serotypes and also has the presence of the two main transmission vector species: *Aedes aegypti* and *Aedes albopictus* (3, 4). While most DENV infections present little or no clinical symptoms, a small proportion of cases progresses to severe forms (1). The variability in clinical presentation of symptoms makes tracking DENV transmission rates difficult among susceptible populations (4). Dengue epidemics show local variations in risk influenced by the densities of mosquito-vector populations due to rainfall, temperature and unplanned rapid urbanization, suboptimal housing conditions, and poor access to clean water (5, 6). Recently, a high correlation has been reported between dengue transmission and the poorest areas belonging to the lowest levels of the socioeconomic stratification (7, 8).

During blood feeding, *Aedes* female mosquitoes inject saliva into host skin to facilitate blood uptake, which initiates a protein-mediated immune response (9). Previous research indicates that mosquito salivary proteins can induce significant antibody responses closely related to the number of bites received by a person. This can then be translated as the intensity of exposure, and could conceivably be used as markers to evaluate vector exposure and dengue transmission risk (10, 11). Certain salivary proteins are known as genus-specific biomarkers, which can help pinpoint vector exposure among host population (12). Specifically, the gSG6 and cE5 proteins are good indicators of human exposure to *Anopheles* mosquito bites while the Nterm-34 kDa peptide allows for evaluation of human exposure to *Aedes* bites (13, 14). Although human exposure does not always present disease transmission, human disease status impacts the antibody response to new exposures (12). Specifically, the D7 family of mosquito salivary proteins has been found to induce higher antibody levels depending on the disease status of the individual (9, 15). Previous research has also indicated that the 38 kDa D7 protein is upregulated in mosquitoes infected with DENV2 (15). Moreover, the D7 immunomodulatory proteins deflect the vertebrate response against biting injury caused during mosquito feeding (16, 17) and potentially favor dengue transmission by recruiting target cells to the bite site (9, 18).

Although considerable research in DENV is focused on pathogen-induced disease severity (19–22), there is a need for studies focused on the role of arthropod vector factors, such as salivary proteins, on DENV infectivity and disease development (23). The objective of this study was to determine whether there is an association between antibody levels against two different *Ae. aegypti* D7 salivary proteins, D7 long (D7L) and D7 short (D7S), and DENV infection in people naturally exposed to *Ae. aegypti* bites in dengue-endemic areas from Colombia. We also evaluated the association between the antibody response against the D7 proteins and factors such as age and living conditions. The likelihood of blood feeding and dengue transmission increases as socioeconomic status decreases due to the proximity of human hosts to vector populations (2, 24, 25). This pilot study represents the first approximation to elucidate any correlation between the antibody response against D7 salivary proteins and the distribution of such response in dengue-endemic areas.

## Materials and Methods

### Study Design and Population

The protocols and methods for this study were reviewed and approved by Los Patios Hospital Ethics Review Board (FGI01-06). Participant’s recruitment was conducted at the Los Patios Hospital (Norte de Santander—Colombia). Patients consulting at the Los Patios hospital for febrile illnesses compatible with dengue were included in the study. In brief, a patient with a “probable dengue” diagnosis and remitted to the laboratory for blood testing is invited to participate. As non-febrile controls, we enrolled non-febrile volunteers accompanying the febrile patients to the laboratory testing if the patient has already agreed to participate in the study. The research objective was explained to each participant and a written informed consent was obtained before collecting samples. A total of 63 participants were enrolled in the study from March to June of 2015. Participants were categorized into three disease status categories: status 1, non-febrile subjects, defined as companions of febrile patients, also considered as dengue exposed subjects but with a negative RT-PCR test for DENV. Status 2, febrile subjects, defined as febrile patients with compatible symptoms to dengue fever but with a negative RT-PCR test for DENV. Status 3, DENV infected subjects, defined as participants with a positive DENV infection confirmed by RT-PCR. DENV infection status was determined by the presence of viral RNA extracted from serum samples according to methods described by Londono-Renteria et al. (10) using DENV serotypes specific primers described elsewhere (26). Briefly, RNA was extracted from the participant’s serum sample using the Quick RNA-Viral kit (Zymo Research). qRT-PCR conditions on the CFX96 Touch™ Real-Time PCR Detection System were: RT Step: 48°C for 5 min, and 95°C for 2 min. Amplification step: (95°C for 15 s, 60°C for 20 s) × 40 cycles.

The living conditions of participants are based on the socioeconomic stratification for the Barrios (Neighborhoods) in the city (Cucuta) according to the Colombian National standards (27). In Colombia, the socioeconomic stratification is divided into six categories, with stratum no. 1 as the lowest and stratum no. 6, the highest. The stratification is based on urban settings characteristics, housing conditions, and access to public utilities (27). In this study, the living conditions of the participants corresponded to three lowest types of strata according to the Colombia’s socioeconomic stratification: Stratum 1 as lower-low, stratum 2 as low, and stratum 3 as upper-low. There was no participant in this study that reported living in any of the higher strata (Stratum 4 or median, stratum 5 or median-high, and stratum 6 or high).

### D7 Recombinant Proteins

The recombinant D7L protein (AAEL006424) NCBI accession#EAT41994, UniProtKB/Swiss-Prot: P18153.2 and the D7S protein [UniProtKB—Q1HRR6 (Q1HRR6_AEDAE)] were produced according to the methods published by Conway et al. (15). Protein concentration was determined using the Thermo Scientific NanoDrop™ (Thermo Fisher Scientific, Wilmington, DE, USA). All ELISA testing was performed using 1 μg/ml as the final protein concentration.

### Antibody Detection by ELISA

The level of human IgG antibodies against mosquito salivary proteins was determined by an indirect ELISA following the methods published by Londono-Renteria et al. (10, 28). Briefly, 96-well ELISA plates (Nunc-Maxisorp, Nalgene Nunc International, Rochester, NY, USA) were coated with 100 μl/well of *Ae. aegypti* salivary gland extract (SGE) or individual D7 recombinant proteins in a final concentration of 0.5 µg/ml of prepared in coating solution (Kierkegaard and Perry Laboratories, Gaithersburg, MD, USA). Serum samples were tested in duplicate in a 1/100 dilution. After washes, plates were incubated with horseradish peroxidase-conjugated goat anti-human IgG (1:1,000) (Abcam, Ab81202) and colorimetric development was obtained using tetra-methyl-benzidine (one-solution micro-well, Gene-Script, Piscataway, NJ, USA). The reaction was stopped with 1 M phosphoric acid and absorbance was measured at 450 nm. Three controls were included on each plate: (1) control blank: two wells without SGE as control for nonspecific induction of color for any of the reagents used in the test; (2) negative control: two wells with SGE but without human serum as control for any nonspecific color induction of the coating antigen; and (3) positive control: 1 control per plate to test plate variation and normalize OD (optical density) values. IgG antibody levels are reported as ΔOD = Average patient OD value (duplicate) − Blank OD value (28).

### Statistical Analysis

Differences in the antibody levels (ΔOD) between two groups (i.e., IgG levels in non-febrile subjects versus IgG levels in DENV infected subjects) were measured by the non-parametrical Mann–Whitney *U* test; while Spearman rank’s correlation test was used to measure association between variables within and between groups (i.e., age and IgG antibody levels). We also conducted a three-way analysis of variance (ANOVA) to compare the main and partial influences of age, disease status, and living conditions (as socioeconomic stratification) on the IgG antibodies against D7S and D7L proteins.

Cluster analysis of data using *k*-mean method, suggests categorizing the participants roughly into four age groups: group 1, ages 1–19 years old; group 2, ages 20–39 years old; group 3, ages 40–59 years old; and group 4 with individuals older than 60 years. In addition to determining whether a factor has a statistically significant difference in the mean level of IgG (D7L or D7S), we measured the effect size of such factor by partial eta-squared. These statistics explain the proportion of variance accounted by an independent effect. Partial eta-squared shows better consistency than eta-squared for measuring effect size in experiments with different designs (29). The effect size was obtained from and standardized according to Richardson et al. (30), and the following guidelines from Ref. (29):

- η^2^ < 0.059 represents a small effect size
- 0.059 ≤ η^2^ < 0.138 represents a medium effect size
- η^2^ ≥ 0.138 represents a large effect size.

Statistical analyses were performed using R 3.4.

## Results

**Figure 1:**
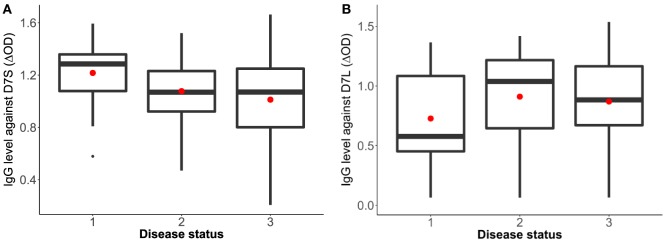
Human IgG antibody levels against *Aedes aegypti* D7 short **(A)** and D7 long **(B)** salivary proteins and disease status. Status 1: non-febrile, non-dengue virus (DENV) infected control subjects (*n* = 12), status 2 febrile subjects (*n* = 12), and status 3: DENV infected subjects (*n* = 37). The red dot indicates the mean and the bars show the first and third quartiles with median as the centerline.

Sixty-three volunteers were included in this pilot study (28 females and 35 males) with an age range between 1 and 82 years old (mean age = 22.6). The analysis of the antibody response against the D7 proteins and the disease status (non-febrile, febrile, and dengue positive) indicates a higher antibody response against the D7S in non-febrile subjects in comparison to DENV-infected subjects (*p* = 0.0742) while non-febrile subjects presented slightly lower antibody levels against the D7L than the other groups (*p* = 0.3435) (Figure 1).

**Figure 2:**
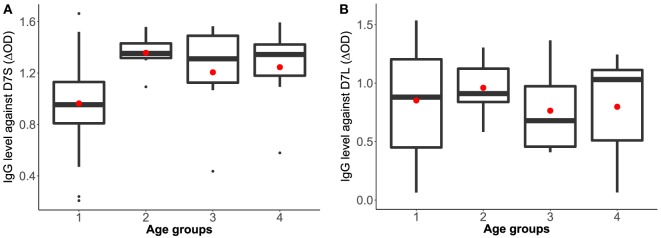
*Aedes aegypti* D7 short **(A)** and D7 long **(B)** salivary proteins and IgG antibody levels represented by age groups. Group 1: ages 1–19 years old (*n* = 41), group 2: ages 20–39 years old (*n* = 7), group 3: ages 40–59 years old (*n* = 6); and group 4: individuals older than 60 years (*n* = 7). The red dot indicates the mean and the bars show the first and third quartiles with median as the centerline.

Our analysis of the antibody levels by age categories revealed a significant positive correlation between age and IgG antibodies against D7S (*r*^2^ = 0.3820, *p* = 0.0024) (Figure 2). We also observed a positive correlation between age and IgG antibodies against D7L; however, this association was not statistically significant (*r*^2^ = 0.1067, *p* = 0.4132).

Table 1 shows the results of three analyses with the test statistics *F*-ratio, the statistical significance with α = 0.05 and also effect size on the analysis. In order to ease the reading of the table, Cohen’s guidelines for the effect size are included (29). The main effect for age yielded an *F*-ratio of *F*(3, 53) = 4.685 and *p* = 0.005, demonstrating a significant difference between observed means of IgG concentration against D7S among the different age groups. A pairwise *t*-test (with pooled variance) suggested a significant difference between age group 1 (1–19 years old) and the rest of the age groups. This result indicates that the average of individuals in age group 1 exhibits significantly different IgG antibody level against D7S, also observed in Figure 2.

**Table 1:** Analysis of variance table showing the test statistics *F*-ratio, statistical significance with α = 0.05, and effect size reported for each variable (age, disease status, and living conditions) evaluated in D7 short and D7 Long for the total sample size (*n* = 63 participants).

D7	Effect	*F*-ratio (df1,df2)	Statistically significant (*p*-value)	eta-squared	Effect size (Cohen’s guidelines)
D7 short	Age	4.685 (3,53)	Yes (0.005)	0.168	Large
	Disease status	0.450 (2,53)	No (0.634)	0.014	Small
	Socioeconomic strata	0.337 (2,53)	No (0.710)	0.012	Small

D7 long	Age	0.295 (3,53)	No (0.829)	0.022	Small
	Disease status	0.747 (2,53)	No (0.479)	0.038	Small
	Socioeconomic strata	1.149 (2,53)	No (0.325)	0.041	Small

*The numerator and denominator degrees of freedom is shown by df1 and df2*.

**Figure 3:**
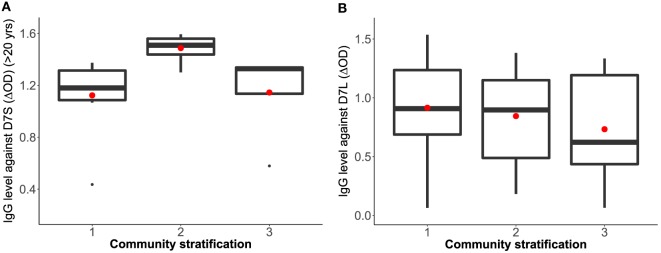
Correlation analysis between *Aedes aegypti* D7 short **(A)** and D7 long **(B)** salivary proteins and living conditions represented as socioeconomic stratification. Stratum 1: lower-low conditions (D7S *n* = 8, D7L *n* = 23), stratum 2: low conditions (D7S *n* = 8, D7L *n* = 26), and stratum 3: upper-low conditions (D7S *n* = 4, D7L *n* = 12). Notice that D7S graphic excludes participants under 20 years old in the analysis.

To isolate the effects of age group 1 in the IgG antibody level against D7S, we excluded individuals under 20 years old and repeated the analysis (Table 2). Furthermore, when age group 1 was excluded, we found that living conditions (socioeconomic stratification) are a statistically significant factor (α = 0.045) in the variability of levels of IgG against D7S. The analysis of the antibody response against the D7S protein for individuals older than 20 years showed higher antibody levels in participants living in stratum 2 (low living conditions) (Figure 3). D7L antibody levels show similarity across the evaluated three socioeconomic strata.

**Table 2:** Analysis of variance table showing the test statistics *F*-ratio, statistical significance with α = 0.05, and effect size reported for each variable (age, disease status, and living conditions) evaluated in D7 short excluding age group 1 (participants under 20 years old).

	Treatment	*F*-ratio (df1,df2)	Statistically significant (*p*-value)	eta-squared	Effect size (Cohen’s guidelines)
D7 short (age > 20)	Age	0.523 (2,10)	No (0.538)	0.0151	Small
	Disease status	0.193 (2,10)	No (0.788)	0.094	Medium
	Socioeconomic strata	3.975 (2,10)	Yes (0.031)	0.379	Large

*The numerator and denominator degrees of freedom is shown by df1 and df2*.

**Figure 4:**
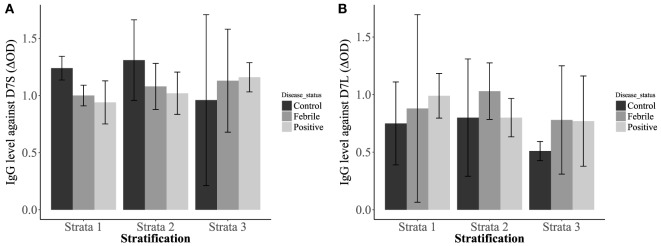
Interaction plots showing the concentrations of IgG antibodies against D7 short **(A)** and D7 long **(B)** when evaluated against the patient disease status: non-febrile subjects (control) (*n* = 12), febrile subjects (*n* = 12), and dengue virus positive subjects (*n* = 37) and living conditions [stratum 1: lower-low conditions (*n* = 23), stratum 2: low conditions (*n* = 26), and stratum 3: upper-low conditions (*n* = 12)].

Our analysis also revealed significantly higher IgG antibody levels in non-febrile patients living in strata 1 and 2 against D7S (Figure 4A). However, the socioeconomic stratum 3 displayed higher IgG antibodies against D7S for febrile and DENV positive participants. Although none of the factors evaluated for D7L showed statistical significance, the effects of disease status and living conditions are higher than age effects (Table 1). Our tests also revealed that D7L is generally higher in febrile patients than non-febrile subjects (control status) (Figure 4B).

## Discussion

Models incorporating anti-salivary protein antibody levels have been shown to be powerful and sensitive biomarkers for a direct and accurate evaluation of the human exposure to mosquito bites (12, 31, 32). Moreover, the detection of increased levels of anti-vector saliva could be pointed as an epidemiological marker of infection and also a suitable indicator of clinical immunity in endemic regions (12). In the case of dengue, it has been shown that antibodies against SGE of *Ae. aegypti* can work successfully as markers of mosquito exposure (12, 33, 34). Furthermore, our previous studies indicate that in dengue-endemic regions, individuals infected with DENV had significantly higher antibody titers to total *Aedes* SGE than non-DENV infected patients (10, 35). Although the whole SGE has been widely used as an approximation to evaluate exposure, SGE enhances the probability of cross-reactivity that may impair the evaluation of the exposure to different vector species (12). This cross-reactivity is caused by different antigens, such as conserved mosquito salivary components, which appear similar to the host molecules or to other arthropod vectors of human disease (36–38). Therefore, the use of species-specific proteins will allow to better calculate dengue transmission and *Aedes*–human contact dynamics. Thus, the finding of a potential “infective bite” marker would significantly advance the protocols available to study the epidemiology of vector-borne febrile illness exposure and help in the design of recommendation guidelines to travelers around the world.

The mosquito D7 salivary proteins were shown to bind biogenic amines, which are important mediators of inflammation, allergy and vascular permeability, and vascular tone (39); consequently, D7 proteins were identified as promising biomarkers of exposure to *Aedes* bites (33, 34). Several members of the D7 protein family are upregulated in DENV infected mosquitoes, and when directly interact with virions, cause infection inhibition (15, 40); however, previous study suggests that, in the human host, the presence of anti-D7 antibodies may be linked to enhanced disease severity (35).

Our analysis showed higher IgG levels against D7L in DENV positive and febrile patients as compared to non-febrile subjects. Previous studies demonstrate that individuals who are exposed to mosquitoes have higher levels of anti-D7L antibodies and the presence of anti-D7 antibodies has been linked to disease severity (15, 35, 40). Conversely, anti-D7 antibodies may prevent mosquitoes from efficient blood feeding, which may enhance disease transmission and disease severity (15). Since D7L is upregulated in infected mosquitoes, our results are consistent with showing that high salivary protein concentrations correlate to higher induction of antibodies in the human host. Thus, the characterizing the factors involved in the vector saliva–virus–host interactions will lead to the development of better strategies to limit disease transmission. The small sample size is one limitation of the current study and larger studies will be necessary to fully understand the role of D7L antibodies and their potential as biomarkers for risk of DENV infection.

Historically, the highest incidence of dengue disease in Colombia was in individuals between 15 and 44 years of age (7). However, in the last decade, the highest rates of occurrence for dengue infections have shifted to younger populations from 4 to 14 years of age (3). Our results indicate that the IgG response to D7S was lower in young populations (under 19 years of age). There was a large effect size in the age variable and a substantial difference in the sample size between age group 1 and the other three groups. Our research further suggests that anti-D7 IgG response can separate both the adaptive condition of exposures in the older population and recent exposure with increased antibody titers in younger populations.

In Colombia, a socioeconomic stratification is a defined classification of the residential real estate by household conditions, which are reflected on differential charges of domiciliary utilities. The classification in any of the six strata is an approximation to the hierarchical socioeconomic difference, from poverty (1, 2, 3) to wealth (4, 5, 6) (DANE-Colombia). In this way, those who have more economic capacity will pay more for public services and contribute so that the lower strata can obtain the services at affordable rates (DANE-Colombia). As a result of this classification system, in Colombia, it is possible to measure the impact to society from shifts in population densities, political, and economic policies as well hazards to human health such as those posed by vector-borne diseases. In the case of dengue, it has been shown that the spatial distribution and impact of the disease are influenced by environmental and socioeconomic factors such as education, occupation, income, population density, and livelihoods (7, 8, 25, 41, 42). Urban space and housing settings are also important dimensions to impacting the dynamics of dengue outbreaks (42). However, the socioeconomic variables seem to be context-dependent, scale-specific, and mainly driven by data availability (7). Studies in Thailand (42), Saudi Arabia (43), and Brazil (24, 25) showed a strong positive association between dengue fever cases and socioeconomic factors. Those studies reveal that the prevalence of dengue infection is significantly higher in deprived areas (lower strata) in comparison with intermediate and privileged areas (upper strata). Therefore, dengue risk is inversely related to the socioeconomic status. Moreover, in deprived areas, the majority of children had already been exposed to DENV by the age of 5 years (4, 24, 25, 41, 42). Although, in this study, we only included the three lower socioeconomic strata because there are historically more associated with the presence of dengue, we were expecting a clear negative correlation between the IgG antibody levels and the socioeconomic status. However, we see that the antibody levels were significantly higher in the strata 2, although we still do not know the exact reason for this result, we believe it is possible that a confounder causes. One of the factors that may be influencing the results is the way stratification is characterized in the area. For instance, previous studies in Colombia have found heterogeneity in dengue exposure and disease within houses sharing the same stratification (7). In addition, house construction materials, access to the sewage system and vegetation coverage, factors not included within the stratification classification, can differ between houses within the same strata. Also, the presence of plastics or other breeding sites for mosquitoes around the houses may influence (11). Unfortunately, we did collect any individual information on the houses, so we cannot exactly pinpoint where the confounder may be. A larger study where the individual household conditions for each study subject may help to clarify these findings.

The investigation into sociodemographic perspective can provide insights for effective management and prevention approaches in areas of high dengue transmission risk (8); however, data-driven tools must incorporate models, which address the physiology of transmission to better estimate transmission risk (44, 45). The use of biomarkers such as the antibodies against salivary proteins of relevance in virus replication is important to identify critical areas within endemic regions where interventions may have higher impact interrupting virus transmission.

In order to evaluate whether socioeconomic stratifications are significant predictors of DENV risk at an urban scale, further research is needed to explore the presence of other salivary biomarkers. Establishing a database of specific biomarkers that relates DENV risk to socioeconomic factors will provide support for public health strategies to enable better detection in high-risk areas. Our research suggests that identifying an *Aedes-*specific salivary protein as an infective bites biomarker would help to more efficiently identify individuals who have been in contact with an infected vector and, therefore, would help to more accurately evaluate the risk of disease transmission.

## Conclusion

Mosquito saliva plays an important role in vector-borne disease transmission and pathology. Immune responses induced by salivary proteins may also have a profound impact on the clinical presentation of diseases like dengue. The present study is a first step toward being able to use human IgG responses to D7 salivary proteins as a biomarker for exposure to infective *Aedes* bites. We found that age is a significant factor in the level of D7S and pooling data for all age groups eliminates effects of patient status and living conditions. Therefore, by separating individuals by their ages, significant large effect for living condition (living strata) and a medium effect, although non-significant, of patient status were observed. However, further studies are needed to test the sensitivity of the biomarker in epidemic settings where *Ae. aegypti*-borne diseases are emerging or re-emerging. A better understanding of the interactions between the host, the vector, and the virus will be valuable to designing more accurate guidelines in endemic regions where residents are frequently bitten by both uninfected and infected mosquitoes.

## Consent for Publication

The authors have no affiliations that represent a conflict of interest regarding this manuscript.

## Availability of Data and Materials

The datasets used and/or analyzed during the current study are available from the corresponding author upon reasonable request.

## Ethics Statement

Protocols and research methods were reviewed and approved by Universidad de Pamplona and the Ethics Review Board of Hospital Erasmo Meoz. The investigation was explained to each individual, and a written informed consent was obtained from each participant or their legal guardian before collecting samples. Blood samples were collected in compliance with the regulations on ethics of research in human participants for Colombia and the United States.

## Author Contributions

BL-R: experimental design and manuscript writing. MC, ND, and TC: recombinant protein synthesis. HS and MJ-D: experimental design, statistical analysis, and manuscript writing. PR-L and TC: manuscript writing.

## Conflict of Interest Statement

The authors declare that the research was conducted in the absence of any commercial or financial relationships that could be construed as a potential conflict of interest.

## Abbreviations

DENV, dengue virus; D7S, protein D7 short; D7L, protein D7 long; SGE, salivary gland extract; ΔOD, delta optical density.
